# C-Kit Is Essential for Vascular Smooth Muscle Cell Phenotypic Switch In Vitro and In Vivo After Injury

**DOI:** 10.3390/cells14201641

**Published:** 2025-10-21

**Authors:** Chiara Siracusa, Giovanni Canino, Mariangela Scalise, Fabiola Marino, Loredana Pagano, Gianluca Santamaria, Annalaura Torella, Salvatore De Rosa, Daniele Torella, Eleonora Cianflone

**Affiliations:** 1 Department of Medical and Surgical Sciences, Magna Graecia University, 88100 Catanzaro, Italy; 2 Department of Experimental and Clinical Medicine, Magna Graecia University, 88100 Catanzaro, Italy; 3 Interdepartmental Center of Services (CIS), Omics Sciences and Biobank, “Magna Græcia” University, 88100 Catanzaro, Italy; 4 Department of Experimental Medicine, University of Campania “L. Vanvitelli”, 80138 Naples, Italy

**Keywords:** c-Kit, vascular smooth muscle cells, phenotypic switch, senescence, neointima formation

## Abstract

Pathological vascular remodeling—central to restenosis, atherosclerosis, and vasculo-proliferative diseases—depends on the phenotypic switching of vascular smooth muscle cells (VSMCs) from a quiescent, contractile state to a synthetic, proliferative program. Although the receptor tyrosine kinase c-Kit is implicated in proliferation, migration, and tissue repair, its role in VSMC plasticity has yet to be fully understood. Using c-Kit haploinsufficient mice subjected to right carotid artery ligation (CAL) and primary aortic VSMC cultures, we show that c-Kit is required for the contractile-to-synthetic transition. In vitro, c-Kit haploinsufficiency halved c-Kit expression, reduced 5-bromo-2′-deoxyuridine (BrdU) incorporation, and blunted platelet-derived growth factor BB (PDGF-BB)-induced repression of contractile genes. c-Kit–deficient VSMCs exhibited a senescence program with increased p16^INK4a^/p21 expression and upregulated senescence-associated secretory phenotype (SASP) mediators. RNA-Seq of carotid arteries 7 days post-ligation revealed that wild-type arteries activated cell-cycle pathways and suppressed contractile signatures, whereas c-Kit-deficient carotid arteries failed to fully engage proliferative programs and instead maintained contractile gene expression. At 28 days post CAL in vivo, c-Kit haploinsufficiency produced markedly reduced neointima, fewer Ki67+ VSMCs, more p16^INK4a^+ cells, and impaired re-endothelialization. Because progenitor-to-VSMC differentiation contributes to remodeling, we tested adult cardiac stem/progenitor cells (CSCs) as a model system of adult progenitor differentiation. Wild-type CSCs efficiently generated induced VSMCs (iVSMCs) with appropriate smooth-muscle gene upregulation; c-Kit–deficient rarely did so. Restoring c-Kit with a BAC transgene rescued both the smooth-muscle differentiation and proliferative competence of c-Kit-deficient iVSMCs. Collectively, our data identified c-Kit as a gatekeeper of reparative VSMC plasticity. Adequate c-Kit enables progenitor-to-VSMC commitment and the expansion of newly formed VSMCs while permitting injury-induced proliferation and matrix synthesis; reduced c-Kit locks cells in a hypercontractile, senescence-prone state and limits neointima formation. Modulating the c-Kit axis may therefore offer a strategy to fine-tune vascular repair while mitigating pathological remodeling.

## 1. Introduction

Cardiovascular diseases (CVDs) including myocardial infarction, stroke, and coronary artery disease are among the leading causes of mortality worldwide, accounting for ~30% of global deaths [1]. These conditions arise primarily from the obstruction or dysfunction of blood vessels [2].

Vascular smooth muscle cells (VSMCs), located in the tunica media, provide structural and functional support to maintain vascular homeostasis. By regulating vasoconstriction and vasodilation, they help preserve blood pressure and flow [3]. Under physiological conditions, VSMCs proliferate very slowly and display minimal synthetic activity. They express contractile proteins such as α-SMA (smooth muscle actin), MYH11 (smooth muscle myosin heavy chain 11), transgelin (SM22α), myocardin, and calponin [4,5]. Despite their differentiated state, VSMCs are not terminally differentiated cells and retain notable plasticity in response to environmental cues and extracellular signals. Following vascular injury, they contribute to neointima formation [6]. In this context, VSMCs are activated and migrate from the media to the intima [7], shifting from a quiescent, highly differentiated state to a synthetic, proliferative phenotype. This switch is characterized by the downregulation of contractile markers and the upregulation of genes associated with migration, proliferation, fibrosis, and inflammation [6,7,8,9,10]. Decreased expression of VSMC-selective markers has been widely documented in animal models of vascular injury/restenosis and in human atherosclerotic lesions [5,11,12], alongside increased VSMC proliferation and migration [13]. Beyond the modulation of medial VSMCs, neointimal growth also involves progenitors—resident adventitial/perivascular cells (e.g., c-Kit+/Sca-1^+^) and context-dependently, circulating/bone-marrow–derived cells (e.g., c-Kit+/Sca-1^+^) [14,15]. These progenitors differentiate toward SMC-like lineages while pre-existing medial VSMCs undergo a contractile-to-synthetic switch, forming a continuum: progenitor-to-VSMC differentiation on one end and dedifferentiation of mature VSMCs on the other, with paracrine signals from progenitors further reinforcing VSMC phenotypic change [15]. Although neointima formation is part of normal vascular repair, excessive growth can cause luminal occlusion and lead to ischemia or vascular insufficiency [16]. Elucidating the molecular signals that govern the contractile-to-synthetic transition is therefore critical for therapeutic development.

Multiple studies implicate the receptor tyrosine kinase KIT (c-KIT) in VSMC phenotypic modulation [17,18,19]. c-Kit, a class III receptor tyrosine kinase, regulates diverse biological processes including hematopoiesis, stem-cell maintenance, gametogenesis, melanogenesis, migration, cell survival, and proliferation [20,21]. It also contributes to cardiac formation, maintenance, and homeostasis as well as responses to myocardial injury and regeneration [22,23,24,25]. Loss-of-function mutations in Kit cause anemia due to reduced erythropoiesis, the absence of melanocytes with a white coat color in mutant mice, infertility, and impaired tissue regeneration after injury in multiple organs [26]. In the vasculature, c-Kit activation after injury has been reported to trigger signaling events that promote the VSMC synthetic/proliferative phenotype [18,27]. However, its precise contribution to neointima formation and injury responses remains unresolved. On the other hand, consistent with a protective role, SMC-specific loss of c-Kit increased plaque burden, necrotic core size, and foam-cell–like features in hyperlipidemic mice, whereas preserving c-Kit signaling in VSMCs limited atherogenesis [28].

Here, we investigated the role of c-Kit in VSMC phenotypic switching using a transgenic mouse model carrying a haploinsufficient c-Kit allele subjected to right carotid artery ligation (CAL). Integrating in vitro studies in primary murine aortic VSMCs with in vivo analyses after CAL, we identified c-Kit as a critical regulator of VSMC proliferation, migration, and differentiation. We combined (i) primary mouse aortic VSMCs challenged with platelet-derived growth factor BB (PDGF-BB), (ii) a right carotid artery ligation model with RNA-Seq at day 7 and morpho-functional analyses at day 28, and (iii) progenitor-to-VSMC differentiation assays with genetic rescue to test whether the c-Kit dosage controls the contractile-to-synthetic switch and reparative proliferation. We identified c-Kit as a dosage-dependent regulator that constrains senescence and enables progenitor commitment and VSMC expansion following injury.

## 2. Materials and Methods

### 2.1. Mouse Model and Right Carotid Artery Ligation

All animal experimental procedures were approved by Magna Graecia Institutional Review Boards on Animal Use and Welfare and performed according to the Guide for the Care and Use of Laboratory Animals from Directive 2010/63/EU of the European Parliament. All animals received human care, and all efforts were made to minimize animal suffering.

Constitutive 8–10-week-old c-Kit haploinsufficient mice [25,29,30] and C57BL/6J wild-type (WT) mice (Jackson Labs, stock number 000664) were used. Clearly, our c-Kit haploinsufficient model reduced c-Kit in multiple lineages and not SMC specifically. Animals were housed under controlled conditions at 25 °C, 50% relative humidity, and a light cycle of 12 h (6:00 a.m.–6:00 p.m.) and 12 h of darkness, with water and food available ad libitum. Before each invasive procedure, mice were anesthetized via intraperitoneal injection of ketamine (100 mg/kg) and xylazine (5 mg/kg) or by the inhalation of isoflurane (Iso-Vet, Healthcare, Aurora, ON, Canada).

To develop a vascular injury model, blood flow in the right common carotid artery of 8–10-week-old c-Kit haploinsufficient mice (N = 23 animals, 16 male and 7 female) and C57BL/6J WT mice (N = 23 animals, 16 male and 7 female) was reduced. This carotid ligation model induces low-flow injury without hyperlipidemia; thus, lipid-driven inflammation typical of atherosclerosis was not modeled here. Briefly, mice were placed in an induction chamber with isoflurane 1.5% in 98.5% oxygen and maintained by the continuous inhalation of isoflurane through a nasal tube. The artery was ligated in two loops using a 7-0 monofilament polypropylene suture proximal to the internal-external carotid bifurcation. Surgical procedures were conducted aseptically, and the surgical incision was closed with a 3-0 non-absorbable silk suture. Carotid samples were collected at 7 days (N = 9 WT and N = 9 c-Kit haploinsufficient male mice) and 28 days (N = 14 WT and N = 14 c-Kit haploinsufficient mice; 7 male and 7 female) after occlusion. To minimize the number of animals used, the contralateral carotid artery of each animal was used as a control.

### 2.2. Isolation of Primary Murine Aortic SMCs and Maintenance in Culture

Mice were anesthetized and placed on a surgical table in a supine position. The thoracic cavity was opened by cutting the diaphragm. Heart, lungs, and abdominal organs were removed to facilitate aortic isolation. Using sterile scissors, the descending aorta was sectioned along with the surrounding perivascular connective tissue. Aortic tissue was transferred to a 60 mm plate containing wash solution, and the surrounding adipose and connective tissue were carefully removed. The isolated aorta was then transferred to a new 60 mm plate and cut into small rings using a scalpel. The aorta was cultured overnight with 2 mL of DMEM + 15% FBS + PenStrep/Fungizone + 10 mg/mL collagenase at 37 degrees with 5% CO_2_. Primary murine aortic SMCs were maintained in DMEM supplemented with 15% FBS, 1% penicillin/streptomycin, and fungizone under standard cell culture conditions (37 °C, 5% CO_2_ in a humidified incubator). After the first overnight incubation, the medium containing the aortic tissue was pipetted up and down and transferred to a 15 mL tube, then centrifuged for 5 min at 180× *g*. The supernatant was discarded, and the pellet containing the SMCs was resuspended and placed in culture in a new 60 mm plate. After 3–5 days, the medium was changed every 2 days until confluence was reached. Primary cells were used for up to three passages to avoid extensive loss of contractile characteristics. A total of N = 9 WT mice and N = 9 c-Kit haploinsufficient mice were used. For each biological replicate, aortic SMCs were isolated by pooling tissues from N = 3 animals, resulting in three biological replicates per group.

### 2.3. Immunocytochemistry

To determine the purity of the cell culture, isolated primary aortic SMCs were stained with a monoclonal anti-α-smooth muscle actin (α-SMA) antibody. Briefly, SMCs were seeded for 24 h in proper plates. Then, cells were fixed using the Cytofix/Cytoperm solution on ice for 15 min. The fixative was then removed, and the cells were washed twice with PBS. To prevent non-specific antibody binding, a 10% donkey serum for 30 min was used, then cells were incubated overnight at 4 °C with the anti-SMA antibody (1:200, Sigma-Aldrich, St. Louis, MO, USA). The following day, cells were washed three times with PBS-Tween 0.1% and incubated with a secondary antibody Alexa Fluor 488 (1:100, Jackson Immunoresearch, Ely, Cambridgeshire, UK) for 1 h at 37 °C in the dark. After incubation, the cells were washed three times with PBS-Tween 0.1% and stained with DAPI (1 µg/mL) and HOCHEST (10 µg/mL) 1:1 ratio for 10 min at room temperature before being mounted with mounting medium (Vectashield, Vector Laboratories, Newark, CA, USA and analyzed using fluorescence microscopy (Leica TCS SP8, Wetzlar, Germany).

### 2.4. Immunohistochemistry

For the immunohistochemical analysis, the carotids of mice were isolated 28 days after ligation and fixed with 4% paraformaldehyde. The samples were then embedded in Optimal Cutting Temperature Compound (OCT) and the tissues were sectioned into 5 µm cross-sections. To detect SMA, Ki67, p16, and CD31-positive SMCs, the following primary antibodies were used: anti-SMA (dilution 1:200; Sigma-Aldrich, St. Louis, MO, USA), anti-CD31 (dilution 1:50; Santa Cruz, Dallas, TX, USA), and anti-p16 (dilution 1:20; Abcam, Cambridge, UK). Primary antibodies were detected by the respective secondary antibodies (anti-mouse IgG or anti-rabbit IgG; dilution 1:100; Jackson Immunoresearch, Ely, Cambridgeshire, UK). Nuclei were counterstained with DAPI. Sections were examined by confocal microscopy (Leica TCS SP8, Wetzlar, Germany). Neointima formation in carotid sections following vascular injury was assessed by hematoxylin and eosin (H&E) staining (Bioptica, Milan, Italy). The content of collagen and muscle fibers after vascular damage was evaluated through Masson’s trichrome staining. The immunohistochemical analysis of cellular proliferation following vascular injury was performed by incubating the sections with the anti-Ki67 antibody (dilution 1:50, DAKO, Santa Clara, CA, USA). Positive reactions were visualized using a horseradish peroxidase polymer complex and 3,3′-diaminobenzidine tetrahydrochloride (DAB) chromogen (EnVision + Dual Link System-HRP; DAKO, Santa Clara, CA, USA). The sections were then counterstained with hematoxylin and examined under optical microscopy (DMI3000B; Leica, Wetzlar, Germany). The number of Ki67-positive cells was expressed as the percentage of total nuclei.

### 2.5. 5-Bromo-2′-Deoxyuridine (BrdU) Incorporation Assay

To evaluate the proliferative activity of aortic SMCs in vitro, a BrdU-based assay was performed. Cells were first placed for 48 h under serum-free medium conditions to synchronize their cell cycle. Then, 10 μM BrdU was administered twice to cell plates, and cells were analyzed at 12 h (T12) and 24 h (T24) from treatment. To visualize BrdU-positive cells, the latter were incubated with anti-BrdU antibody using the kit and following the manufacturer’s instructions. Nuclei were stained with DAPI, and cells were analyzed using fluorescence microscopy. The number of BrdU+ cells was expressed as a percentage of the total cell nuclei. Each treatment was performed in technical triplicate within each biological replicate.

### 2.6. Cell Proliferation Assay

Aortic SMCs were plated in a 24-well plate at a concentration of 2 × 10^4^ cells/well. Cells were placed for 48 h under the serum-free medium condition to synchronize their cell cycle. Afterward, cells were counted using a Burker chamber every 24 h for 4 consecutive days.

### 2.7. Flow Cytometry

Cells were harvested and fixed with 4% paraformaldehyde for 10–15 min at room temperature, followed by permeabilization with 0.1–0.2% Triton X-100 in PBS for 10 min. After washing, cells were incubated with an APC-conjugated anti-α-SMA antibody (Abcam, Cambridge, UK) for 30 min at 4 °C in the dark. Unbound antibody was removed by washing with PBS, and cells were analyzed by flow cytometry. Appropriate isotype controls were included for gating and compensation. FACS analysis was performed using FACSCanto II (BD Biosciences, Paramus, NJ, USA), and FlowJo software v10 (Tree Star, Ashland, OR, USA) was used to quantify the percentage of cells expressing the marker of interest.

### 2.8. BAC c-Kit Vector Generation and Transfection in c-kit^w/+^ CSCs

A purified bacterial artificial chromosome (BAC) containing the entire c-Kit gene (BACc-Kit) was generated and transfected into c-Kit^w/+^ cardiac stem cells (CSCs), as previously described [25,29].

### 2.9. Smooth Muscle Cell Differentiation

WT-CSCs and c-kit^w/+^-CSCs were plated in LIF-deprived basic differentiation medium consisting of α-MEM, dexamethasone (1 μM), ascorbic acid (50 μg/mL), and β-glycerophosphate (10 mM; all from Sigma) with 3% FBS (Life Technologies, Carlsbad, CA, USA). The differentiation medium was implemented with TGF-β1 (5 ng/mL), all-trans retinoic acid (atRA; 10^−6^ mol/L, Sigma), and complete differentiation media refreshed every 72 h. Cell differentiation was evaluated at 14 days.

### 2.10. PDGF-BB Cell Treatment

WT-VSMCs and c-kit^w/+^-VSMCs were cultured under standard conditions at 37 °C until reaching approximately 70–80% confluency. The growth medium was then replaced with a serum-free medium for 48 h to produce cellular starvation. WT-VSMCs and c-kit^w/+^-VSMCs were subsequently stimulated with PDGF-BB (20 ng/mL; R&D Systems, Minneapolis, MN, USA) for 24 h.

### 2.11. RNA Extraction and RT-PCR Analysis

RNA was extracted using the TRIzol reagent (Life Technologies, Carlsbad, CA, USA) from cells and tissues according to the manufacturer’s instructions. cDNAs were generated with the High-Capacity cDNA Reverse Transcription Kit (Thermo Fisher Scientific, Waltham, MA, USA). The expression of cell proliferation and senescence markers was evaluated using Taqman and SYBER GREEN PCR (Thermo Fisher Scientific, Waltham, MA, USA). The following markers were analyzed: Tagln1 (Transgelin/SM22α), Acta2 (Actin, alpha 2, smooth muscle), Myh11 (Myosin heavy chain 11), Cnn1 (Calponin 1), Cdkn2a (p16^INK4a^), Cdkn1a (p21), Serpine1 (PAI-1), matrix metalloproteinase-3 (Mmp3), tumor necrosis factor alpha (TNF-α), interleukin 6 (Il6), Ki67, and receptor tyrosine kinase c-Kit (c-Kit). Real-time PCR was performed on a 96-well plate system (Applied Biosystems, Waltham, MA, USA). Relative gene expression was determined using the deltaCT method with StepOnePlus Software v2.3 (Applied Biosystems, Waltham, MA, USA). RNA was normalized to GAPDH, and all reactions were conducted in triplicate.

### 2.12. RNA-Seq Analysis

Carotid samples were collected at 7 days (N = 6 WT and N = 6 c-Kit haploinsufficient mice) after occlusion, and RNA was extracted from tissue using the TRIzol reagent (Life Technologies, Carlsbad, CA, USA) for transcriptome analysis via RNA sequencing. The contralateral carotid from the same mice was used as the control (CTRL, N = 6 for each group). For the RNA-Seq experiment, three carotids were pooled to obtain two biological replicates per group to substantially increase the statistical power, obtaining a reliable assessment of biological variability.

Libraries were generated using depleted RNA obtained from 1 μg of total RNA with a TruSeq Sample Preparation RNA Kit (Illumina, Inc., San Diego, CA, USA) according to the manufacturer’s protocol. All libraries were sequenced on Illumina HiSeq 1000, generating paired-end reads of 100 bp. Following library preparation, transcriptome data were processed to identify sets of upregulated and downregulated genes, grouped with Gene Ontology tools, in different samples.

All FastQ files were quality checked using FastQC software (v0.12.0); then, adapter sequences were removed, and low-quality reads were filtered out using FastQC software (v0.12.0). The resulting high-quality reads were then mapped to the mouse reference genome (GRCm39).

This alignment was performed using HISAT2 2.2.1 (http://github.com/infphilo/hisat2 accessed on 26 May 2025). For the analysis of raw count data, the DESeq2 package (version 1.44.0 https://github.com/thelovelab/DESeq2 accessed on 3 June 2025) in the R programming language (version 4.4.1) was employed using the RStudio integrated development environment (RStudio version 2024.09.0 +375 “Cranberry Hibiscus”). Differential gene expression analysis was conducted by comparing each experimental condition with the respective controls. Gene expression was normalized using the variance stabilizing transformation, centered, and Z-scaled for visualization purposes. For Gene Ontology (GO) analysis of differentially expressed (DE) genes, the DESeq2 package (version 4.4.1) was used to identify relevant biological pathways [31].

Due to repository processing timelines, the accession number for the RNA-seq data is not yet available at time of publication. The data are being deposited in an internationally recognized repository, and the authors will provide the accession upon direct request. 

### 2.13. Statistical Analysis

Data are presented as the mean ± SD. A *p*-value of *p* < 0.05 was considered statistically significant. Significance between groups was determined using the Student’s *t*-test and for multiple comparisons with analysis of variance (ANOVA) using GraphPad Prism version 9.4.0 for Windows (GraphPad Software, version 9.4.0, San Diego, CA, USA).

## 3. Results

### 3.1. Isolation and Characterization of Mouse Aortic SMCs

To investigate the role of c-Kit in the VSMC phenotypic switch, we used a c-Kit haploinsufficient mouse model carrying a c-Kit null allele generated through CRE recombinase knock-in at the c-Kit locus [29,30]. These mice show the typical white (W) spotting mutant phenotype, a coat color depigmentation, generated by c-Kit loss of function. Age- and sex-matched C57BL/6J wild-type (WT) mice served as controls [32,33].

**Figure 1:**
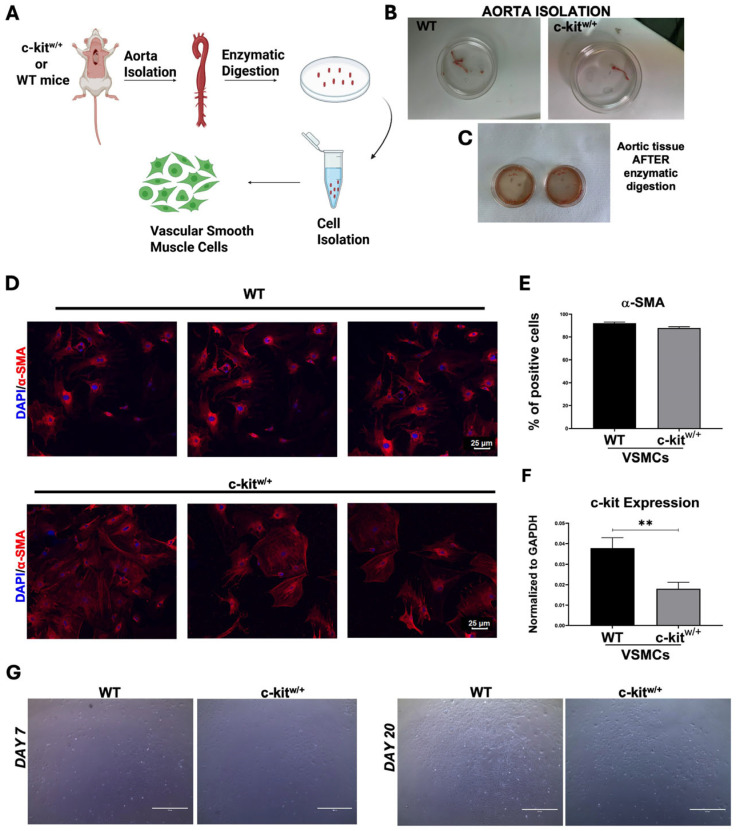
Isolation and characterization of aortic murine SMCs in vitro. (**A**) Schematic representation of smooth muscle cell isolation protocol from WT and c-Kit haploinsufficient mice. “Ci, E. (2025) https://BioRender.com/twgwmq8”. (**B**,**C**) Representative images showing mouse descending aorta isolated and collected in a 60 mm Petri dish in phosphate-buffered saline (PBS) from both mouse models (**B**) and its enzymatic digestion (**C**). (**D**) Representative confocal microscopy images showing α-SMA-positive SMCs in vitro after aortic isolation from WT and c-kit^w/+^ mice (α-SMA, red; nuclei/DAPI/blue). Scale bars = 25 μm. (Representative of n = 3 biological replicates, each performed in triplicate). (**E**) Bar graph showing the percentage of α-SMA-positive SMCs after aortic isolation from WT and c-kit^w/+^ mice (n = 3 technical replicates for each biological triplicate). (**F**) Bar graph showing c-Kit expression in aortic SMCs from WT and c-kit^w/+^ mice (n = 3 technical replicates for each biological triplicate) ** *p* = 0.0044 vs. WT. (**G**) Representative light microscopy images showing the confluence and morphology in vitro of SMCs from WT and c-kit^w/+^ mice after 7 and 20 days from isolation. Scale bars = 200 um, n = 3 technical replicates for each biological triplicate. Data are the mean ± SD.

Aortic VSMCs were isolated from 8–10-week-old WT and c-Kit haploinsufficient mice (hereafter c-kit^w/+^) using a standardized protocol (Figure 1A–C). The thoracic aorta was sectioned and transferred to a 60 mm plate to carefully remove adipose and connective tissue, then cut into small rings and cultured overnight with 2 mL of DMEM + 15% FBS + PenStrep/Fungizone + 10 mg/mL collagenase (Figure 1A–C). The day after, the medium containing the aortic tissue was centrifuged, the pellet resuspended and then placed in culture in a new 60 mm plate (Figure 1B,C). The medium was changed every 2 days until confluence was reached. To verify cell purity in terms of VSMC number obtained from the aortic tissue isolation, immunofluorescence staining for α-smooth muscle actin (α-SMA), the actin isoform that predominates within vascular smooth muscle cells, was performed. Approximately 90% of the isolated aortic VSMCs from both WT and c-kit^w/+^ mice were positive for SMA, indicating a highly enriched VSMC population obtained by the isolation protocol used (Figure 1D,E).

VSMCs from c-kit^w/+^ mice had a 50% decrease of c-Kit mRNA expression compared with VSMCs from the WT mice (Figure 1F). WT-VSMCs reached confluence in plates more rapidly and produced higher cell numbers at both 7 and 20 days compared with c-kit^w/+^ counterparts (n = 3 aortic samples per replicate) (Figure 1G). These data point to a significant growth deficit of VSMCs by c-Kit haploinsufficiency.

### 3.2. c-Kit Haploinsufficiency Affects Mouse Aortic VSMC Proliferation Inducing Cellular Senescence and SASP In Vitro

**Figure 2:**
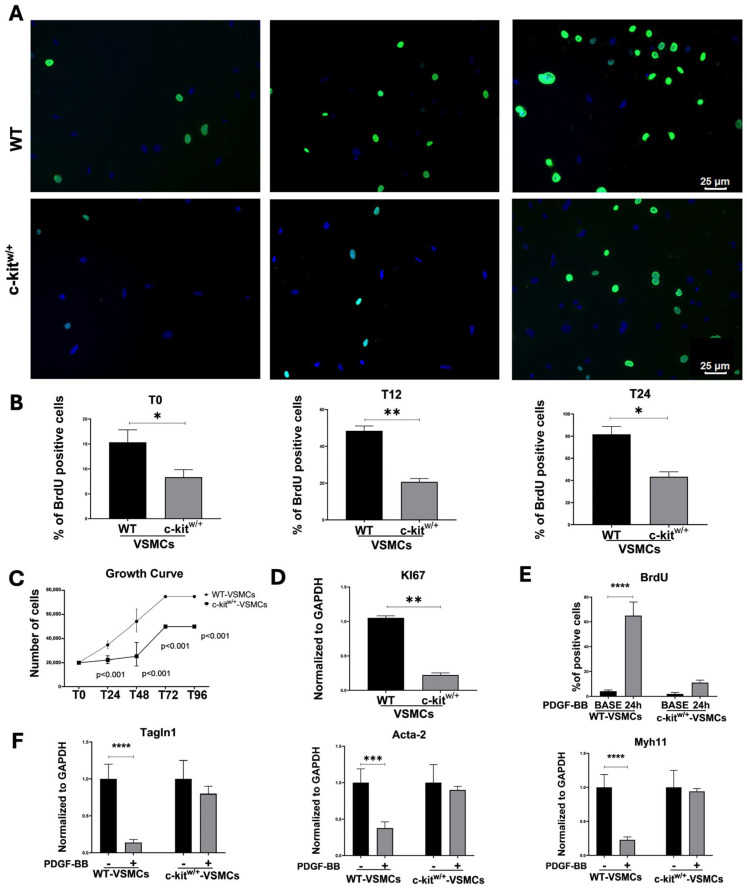
WT- and c-kit^w/+^-VSMCs proliferation in vitro. (**A**) Representative confocal images showing BrdU incorporation in vitro in WT- and c-kit^w/+^-VSMCs at the baseline (T0) (* *p* = 0.0146 vs. WT) and at 12 (T12) (** *p* = 0.0066 vs. WT) and 24 h (T24) (* *p* = 0.0235 vs. WT) from BrdU treatment (BrdU, green; nuclei/DAPI/blue). Scale bars = 25 μm, representative of n = 3 biological replicates, each performed in technical triplicates. (**B**) Bar graphs showing the in vitro percentages of BrdU-positive WT- and c-kit^w/+^-VSMCs at the baseline (T0) and at 12 (T12) and 24 (T24) hours from BrdU treatment (n = 3 technical replicates for each biological triplicate). (**C**) Cell growth curve showing the in vitro proliferation in WT- and c-kit^w/+^-VSMCs at T24, T48, T72, and T96 h. Line plot: circles = WT-VSMCs; squares = c-kit^w/+^-VSMCs; WT counts exceed c-kit^w/+^ by ~2-fold across T24–T96 (two-tailed *t*-test across timepoints, *p* < 0.001). (**D**) Bar graph showing the relative expression of Ki67 in WT- and c-kit^w/+^-VSMCs (n = 3 technical replicates for each biological triplicate). ** *p* = 0.0013 vs. WT. (**E**) Bar graph showing the percentage of BrdU-positive WT- and c-kit^w/+^-VSMC after PDGF-BB treatment (n = 3 technical replicates for each biological triplicate). **** *p* < 0.0001. (**F**) Bar graphs showing the relative expression of Tagln1 (Transgelin/SM22α), Acta2 (Actin, alpha 2, smooth muscle), and Mhy11 (Myosin heavy chain 11) in WT- and c-kit^w/+^-VSMCs (n = 3 technical replicates for each biological triplicate). *** *p* = 0.0009; **** *p* value < 0.0001. Data are the mean ± SD.

To assess the effect of c-Kit haploinsufficiency in VSMC plasticity in vitro, two proliferative assays were conducted on primary mouse VSMCs isolated from the aortas of WT and c-kit^w/+^ mice. BrdU (5-bromo-2′-deoxyuridine), a synthetic nucleoside thymidine analogue that permanently labels proliferating and daughter cells, was administered every 8 h to cultured VSMCs. A twofold higher proliferation rate of WT-VSMCs was observed when compared with the c-kit^w/+^-VSMCs (Figure 2A,B). Interestingly, a higher percentage of proliferative WT-VSMCs was already evident as early as 12 h after treatment, with 50 ± 4% BrdU-positive cells in WT-VSMCs compared with 19 ± 3% in c-kit^w/+^-VSMCs (Figure 2A,B). The higher proliferation rate in WT-VSMCs was persistent for 24 h (81 ± 5 vs. 43 ± 3 in WT-VSMCs and c-kit^w/+^-VSMCs, respectively) (Figure 2A,B). Similar results were obtained performing a growth curve assay at different time points (Figure 2C). A progressive twofold increase in the number of WT-VSMCs was already evident after 24 h (T24) as well as at every other timepoint compared with c-kit^w/+^-VSMCs (Figure 2C). The significant proliferative deficit of c-kit^w/+^-VSMCs was corroborated by qRT-PCR analysis, which revealed a fivefold downregulation in the expression level of the proliferation marker Ki67 in c-kit^w/+^-VSMCs compared with WT-VSMCs (Figure 2D). Thus, these results show that the haploid level of c-Kit in c-kit^w/+^-VSMCs directly alters the proliferative capacity of VSMCs.

An established model for VSMC phenotypic modulation in vitro involves the treatment of VSMCs with platelet-derived growth factor BB (PDGF-BB) to induce smooth muscle gene downregulation and cell proliferation [34,35]. PDGF-BB stimulation of serum-starved WT-VSMCs induced VSMC proliferation measured by BrdU incorporation over 24 h (Figure 2E), reducing the expression of known SMC genes such as Tagln1 (Transgelin, SM22α), Acta2 (Actin alpha 2, smooth muscle), and Mhy11 (myosin heavy chain 11) (Figure 2F). On the contrary, PDGF-BB primed c-kit^w/+^-VSMCs showed a blunted downregulation of SMC genes alongside a deficit in their proliferative activity (Figure 2E,F).

**Figure 3:**
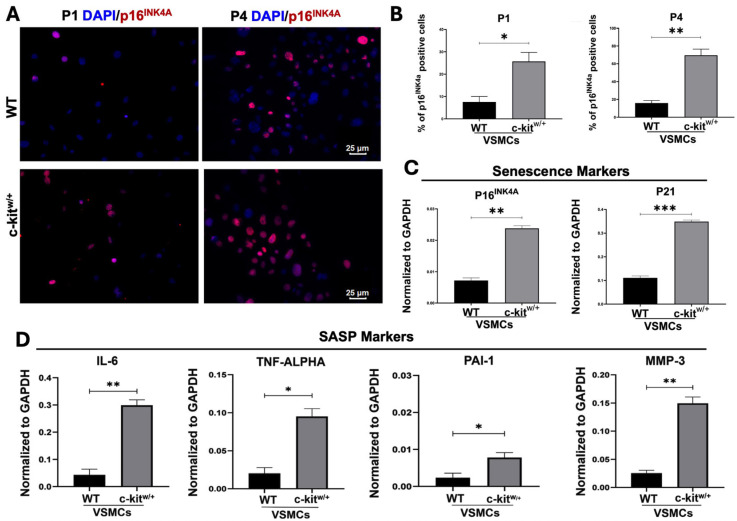
Evaluation of cellular senescence and SASP in vitro in WT- and c-kit^w/+^-VSMCs. (**A**,**B**) Representative confocal images (**A**) and bar graphs (**B**) showing the in vitro percentage of senescent p16^INK4a^–positive WT- and c-kit^w/+^-VSMC mice at two different cellular passages (passage 1 and passage 4, respectively, p1 and p4). p16^INK4a^, red; nuclei/DAPI/blue. Scale bars = 25 μm. (n = 3 technical replicates for each biological triplicate). * *p* = 0.0332 vs. WT; ** *p* = 0.0096 vs. WT. (**C**) Bar graphs showing the relative expression of senescence markers in WT- and c-kit^w/+^-VSMC (n = 3 technical replicates for each biological triplicate). ** *p* = 0.0026 vs. WT; *** *p* = 0.0010 vs. WT. (**D**) Bar graphs showing the relative expression of senescence-associated secretory phenotype (SASP) markers [(interleukin 6 (Il6) (** *p* = 0.0058 vs. WT), tumor necrosis factor alpha (TNF-α) (* *p* = 0.0169 vs. WT), Serpine1 (PAI-1) (* *p* = 0.0114 vs. WT), and matrix metalloproteinase-3 (Mmp3) (** *p* = 0.0034 vs. WT)] in WT- and c-kit^w/+^-VSMC (n = 3 technical replicates for each biological triplicate). Data are the mean ± SD.

To investigate whether the reduced proliferative capacity observed in VSMCs from c-kit^w/+^ mice was due to the acquisition of a senescent phenotype, we analyzed the expression of established senescence markers, including p16^INK4a^. We observed a twofold higher percentage of p16^INK4a^-positive cells in c-kit^w/+^-VSMCs compared with the WT-VSMCs at the baseline (25 ± 3% vs. 7 ± 2% in c-kit^w/+^-VSMCs compared with WT-VSMCS, respectively) (Figure 3A,B). Interestingly, the percentage of p16^INK4a^-positive cells was progressively increased through cell passages in both cell lines, reaching at passage 4 (p4) 66 ± 3% in c-kit^w/+^-VSMCs compared with 16 ± 2% observed in WT-VSMCs (Figure 3A,B). These data suggest that c-kit^w/+^-VSMCs progressively acquired a stable state of cell cycle arrest when primed to grow in culture, corresponding with the development of a cellular senescence phenotype. To confirm this hypothesis, the appearance in cultured VSMCs of cellular senescence markers was assessed by qRT-PCR, which showed a significant threefold upregulation in the expression of p16^INK4a^ (Cdkn2a) and p21 (Cdkn1a) in c-kit^w/+^-VSMCs (Figure 3C). Similarly, genes related to the senescence-associated secretory phenotype (SASP), such as IL-6 (interleukin 6), TNF-alpha (Tumor necrosis factor alpha), PAI-1 (Serpine1) and MMP3 (matrix metalloproteinase-3), were all upregulated in c-kit^w/+^-VSMCs compared with WT-VSMCs (Figure 3D). These genes were respectively 9,8-, 5,7, 4,5-, and 6,5-fold upregulated in c-kit^w/+^-VSMCs compared with WT-VSMCs at the baseline (Figure 3D). Thus, our data demonstrate that c-Kit downregulation in aortic VSMCs, resulting from the c-Kit null allele in c-kit^w/+^ mice, associates with a senescent phenotype by VSMCs acquired when primed to the phenotypic switch in vitro.

### 3.3. c-Kit Haploinsufficiency Affects VSMC Differentiation In Vitro

c-Kit is a key regulator of stem/progenitor-cell fate decisions, but its role in driving VSMC differentiation remains poorly defined. Because VSMC differentiation of vascular and circulating progenitors contributes to neointima formation after vascular injury, clarifying c-Kit’s contribution is biologically relevant. Therefore, we used adult cardiac stem cells (CSCs) as a validated model system of adult progenitors [24] to interrogate the role of c-Kit in VSMC differentiation in vitro.

**Figure 4:**
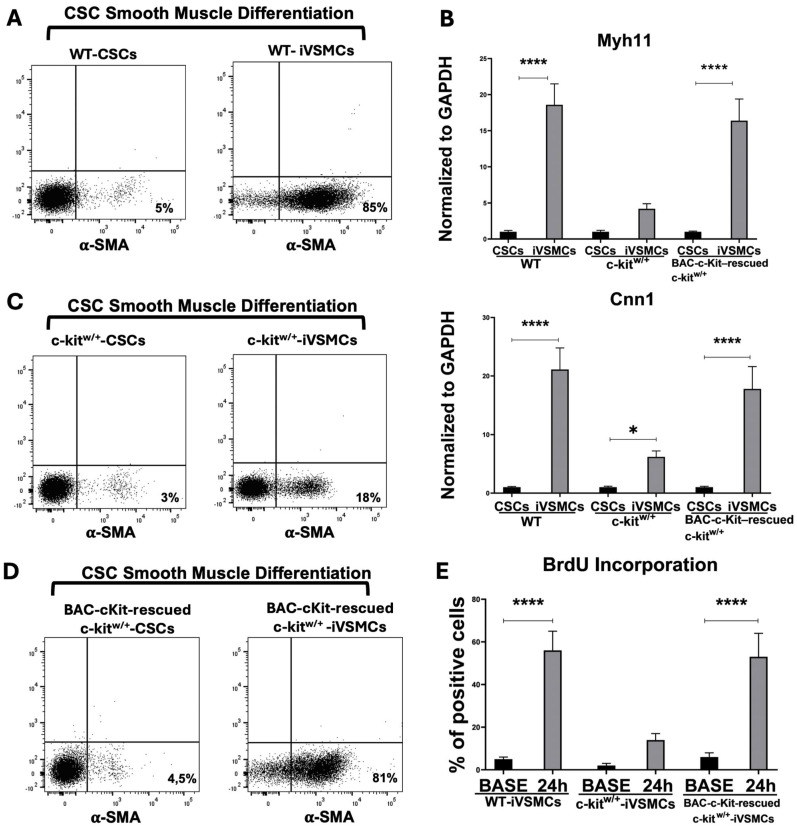
c-Kit haploinsufficiency affects VSMC lineage commitment in vitro. (**A**) Flow cytometry dot plots showing α-SMA expression in WT-CSCs induced to differentiate into smooth muscle cells (n = 3 technical replicates for each biological triplicate). (**B**) Bar graph showing the relative expression of SMC-specific transcripts Myosin heavy chain 11 (Myh11) and Calponin 1 (Cnn1) in WT-, c-kit^w/+^-, and BAC-c-Kit-rescued c-kit^w/+^-induced VSMCs (iVSMCs) 14 days after in vitro smooth muscle lineage commitment (n = 3 technical replicates for each biological triplicate) **** *p* < 0.0001. (**C**,**D**) Flow cytometry dot plots showing α-SMA expression in c-kit^w/+^-CSCs and BAC-c-Kit-rescued c-kit^w/+^-CSCs induced to differentiate into smooth muscle cells (n = 3 technical replicates for each biological triplicate) **** *p* < 0.0001; * *p* = 0.0366. (**E**) Bar graph showing the percentage of BrdU in WT-, c-kit^w/+^-, and BAC-c-Kit-rescued c-kit^w/+^-iVSMCs over 24 h (n = 3 technical replicates for each biological triplicate). **** *p* < 0.0001. Data are the mean ± SD.

When tested for functional smooth muscle differentiation, WT-CSCs homogeneously differentiated into α-SMC-expressing cells (86 ± 10%) within 14 days (hereafter referred to as induced VSMCs, iVSMCs) (Figure 4A). WT-iVSMCs appropriately upregulated SMC-specific transcripts such as Myh11 and Cnn1 (Calponin 1) (Figure 4B). In contrast, c-kit^w/+^-CSCs rarely differentiated into VSMC-positive cells (16 ± 4%) (Figure 4C). Accordingly, SMC-specific transcripts such as Myh11 and Cnn1 were only minimally upregulated in c-kit^w/+^-CSCs compared with undifferentiated CSCs (Figure 4B).

To test whether restoring c-Kit levels could correct the c-kit^w/+^ defects in vitro, we used a c-kit^w/+^ CSC clone rescued by transfection with a BAC construct spanning the entire c-Kit locus [25,29]. In this clone, c-Kit mRNA and protein levels are indistinguishable from WT-CSCs and approximately twofold higher than in the un-transfected parental c-kit^w/+^ CSCs [25]. Notably, BAC-c-Kit transfection rescued the vascular smooth muscle differentiation defect: rescued c-kit^w/+^-CSCs displayed an overall VSMC differentiation potential indistinguishable from WT-CSCs as measured by SMA-expressing cells (83 ± 11%) and smooth muscle gene upregulation (Figure 4B,D).

Based on these data, we assessed the proliferative capacity of WT-iVSMCs, c-kit^w/+^-iVSMCs, and BAC-c-Kit-rescued c-kit^w/+^-iVSMCs. Consistent with results from primary adult VSMC cultures, c-kit^w/+^-iVSMCs showed significantly reduced BrdU incorporation over 24 h when stimulated with 10% serum compared with WT-iVSMCs (Figure 4E). Remarkably, normalization of c-Kit expression by BAC-c-Kit transfection increased proliferation in rescued c-kit^w/+^ iVSMCs to levels indistinguishable from WT-iVSMCs (Figure 4E).

Collectively, these data show that c-Kit is required for VSMC lineage commitment of adult progenitors and for the proliferative competence of newly formed VSMCs.

### 3.4. c-Kit Haploinsufficient Mice Display a Lower Proliferative Activity and an Increased SMC Senescence after Injury

To assess whether c-Kit expression is essential to VSMC phenotypic switch and vascular remodeling after injury in vivo, 8–10-week-old WT and c-kit^w/+^ mice underwent permanent right carotid artery ligation (CAL). Carotid arteries were collected at 7 and 28 days following surgical occlusion. Tissues were either fixed in 4% PFA for immunohistochemical analysis or processed for RNA extraction.

We first performed RNA sequencing on carotid artery tissue harvested 7 days after ligation from WT and c-kit^w/+^ mice. The contralateral uninjured carotids were used as the control. Principal component analysis (PCA) revealed distinct clustering of injured WT (CAL-WT) and c-kit^w/+^ (CAL-c-kit^w/+^) samples, whereas uninjured controls clustered homogeneously (Supplementary Figure S1A). Importantly, transcriptomic analysis showed an enrichment of senescence-related gene expression in uninjured c-kit^w/+^ arteries compared with WT (hereafter UNinj-c-kit^w/+^ and UNinj-WT, respectively) including the upregulation of Cdkn1a (p21). Also, UNinj-c-kit^w/+^ arteries exhibited the downregulation of proliferation-associated genes such as the anti-proliferation factor 2 (BTG2), Wnt Family Member 2 (Wnt2), Wnt Family Member 5A (Wnt5a) and Wnt Family Member 5b (Wnt5b) (Supplementary Figure S1B).

**Figure 5:**
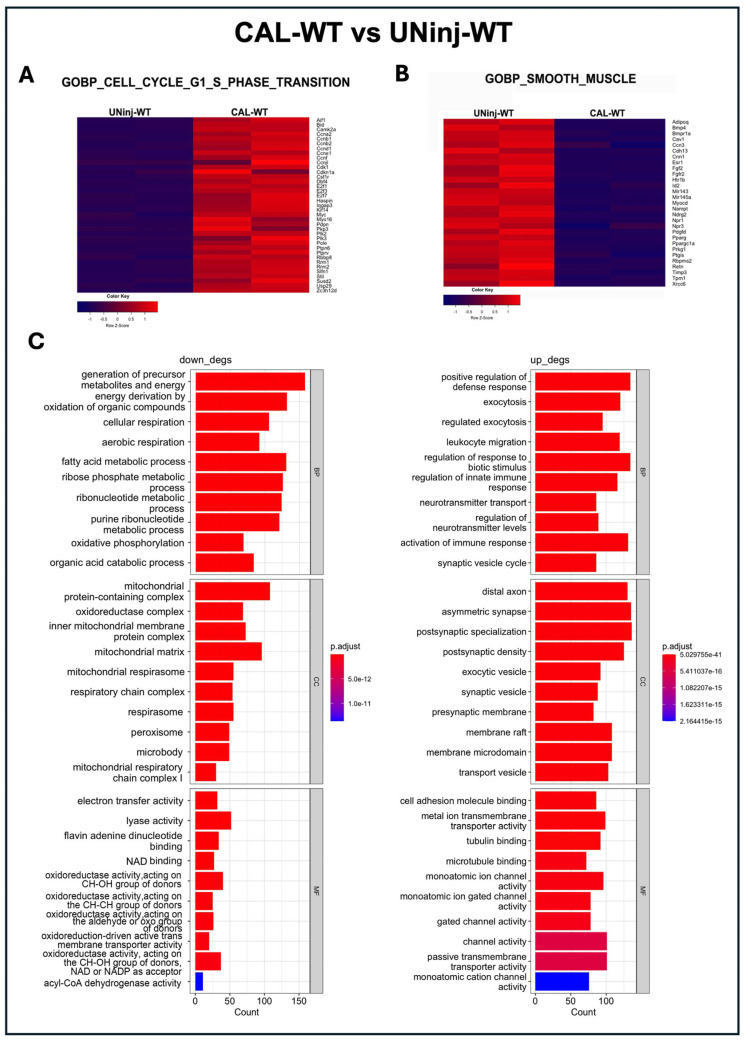
Global transcriptome profiles and gene-expression signatures of WT mice after injury. (**A**) GSEA of G1 and G2 cell cycle-associated genes in CAL-WT compared with UNinj-WT. (**B**) GSEA of smooth muscle-associated genes in CAL-WT samples compared with UNinj-WT. (**C**) Gene ontology respectively showing the downregulated and upregulated pathways in CAL-WT arteries compared with UNinj-WT controls related to specific biological processes (BP) and their relative cellular components (CC) and molecular functions (MF). To perform RNA-Seq analysis, carotids were collected on Day-7: N = 6 WT and N = 6 c-kit^w/+^ carotids; contralateral carotids were used as the controls; n = 3 carotids were pooled to create each of two biological replicates per group.

We then compared carotid samples from CAL-WT and their relative UNinj-WT control. Gene set enrichment analysis (GSEA) of cell cycle-associated genes, particularly those involved in the G1 and G2 phases governing cell growth through the regulation of cell size, protein synthesis, and the production of mitotic components, revealed a higher proliferation in CAL-WT compared with UNinj-WT (Figure 5A). Concurrently, genes related to VSMC differentiation and specification were downregulated in CAL-WT samples compared with the UNinj-WT control (Figure 5B), supporting the notion that VSMC phenotypic switching upon injury in vivo is associated with the reduced expression of canonical smooth muscle markers in favor of a proliferative gene program.

Functional enrichment analyses further indicated a downregulation of pathways related to biological processes (BP) involved with cellular respiration, oxidative phosphorylation, and mitochondrial activity in CAL-WT arteries compared with the UNinj-WT controls, reflecting a change in the metabolic demands of proliferating VSMCs. The cellular components (CC) identified in this comparison were predominantly associated with mitochondrial activity, specifically the mitochondrial respiratory chain, with all super-complexes contributing to mitochondrial processes (Figure 5C).

The relative molecular functions (MF) were accordingly related to energetic metabolism and energy production (Figure 5C). This finding is consistent with the evidence that contractile VSMCs in uninjured healthy arteries depend on mitochondrial-derived metabolic energy to sustain contraction and relaxation, processes that are essential for regulating vascular diameter, airway tone, and motility. Conversely, immune-response pathways were all upregulated in CAL-WT arteries compared with the UNinj-WT controls (Figure 5C).

**Figure 6:**
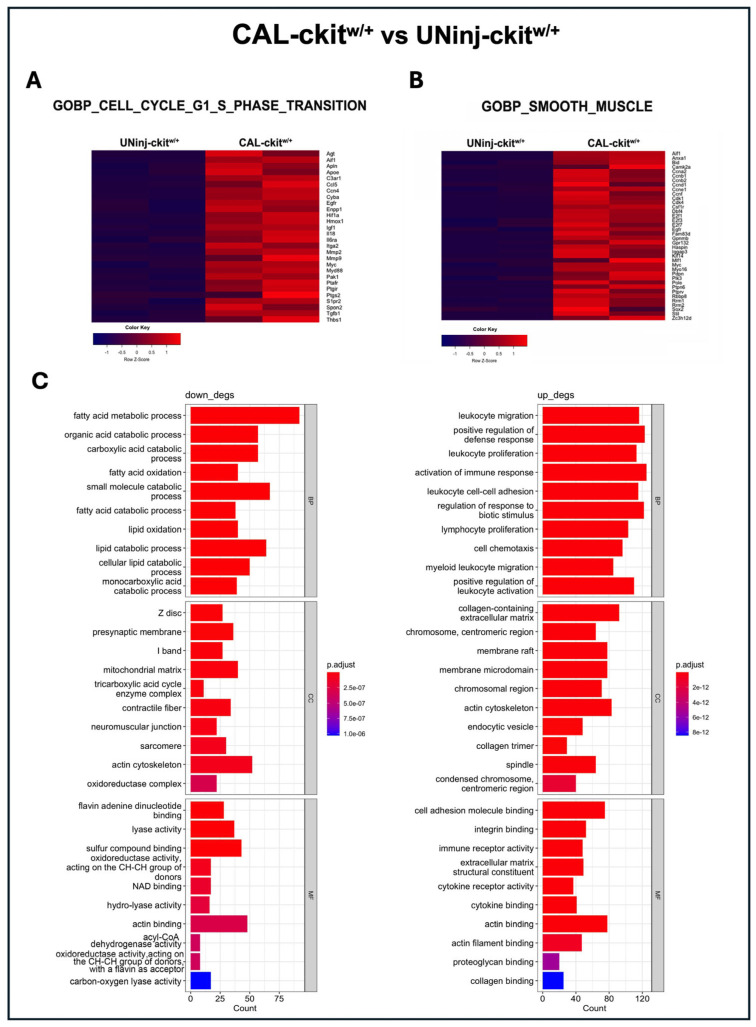
Global transcriptome profiles and gene expression signatures of c-kit^w/+^ mice after injury. (**A**) GSEA of G1 and G2 cell cycle-associated genes in CAL-c-kit^w/+^ versus UNinj-c-kit^w/+^. (**B**) GSEA of smooth muscle-associated genes in CAL-c-kit^w/+^ versus UNinj-c-kit^w/+^. (**C**) Gene ontology respectively showing the downregulated and upregulated pathways in CAL-c-kit^w/+^ arteries compared with the UNinj-c-kit^w/+^ controls and their specific biological processes. To perform RNA-Seq analysis, carotids were collected at Day-7: N = 6 WT and N = 6 c-kit^w/+^ carotids; contralateral carotids were used as controls; n = 3 carotids were pooled to create each of two biological replicates per group.

In the comparison, CAL-c-kit^w/+^ versus UNinj-c-kit^w/+^, arterial injury upregulated genes related to cell proliferation but at a significant lesser level than the CAL-WT arteries (Figure 6A). Accordingly, smooth muscle cell markers (e.g., actin filament binding) were upregulated after damage in UNinj-c-kit^w/+^, indicating a persistent regulation of the contractile response as opposed to the complete phenotypic switch in WT arteries (Figure 6B). When analyzing the downregulated biological processes in the comparison CAL-c-kit^w/+^ versus UNinj-c-kit^w/+^, a similar involvement of the mitochondrial response to injury was observed (Figure 6C), as already shown in the comparison between CAL-WT versus UNinj-WT.

**Figure 7:**
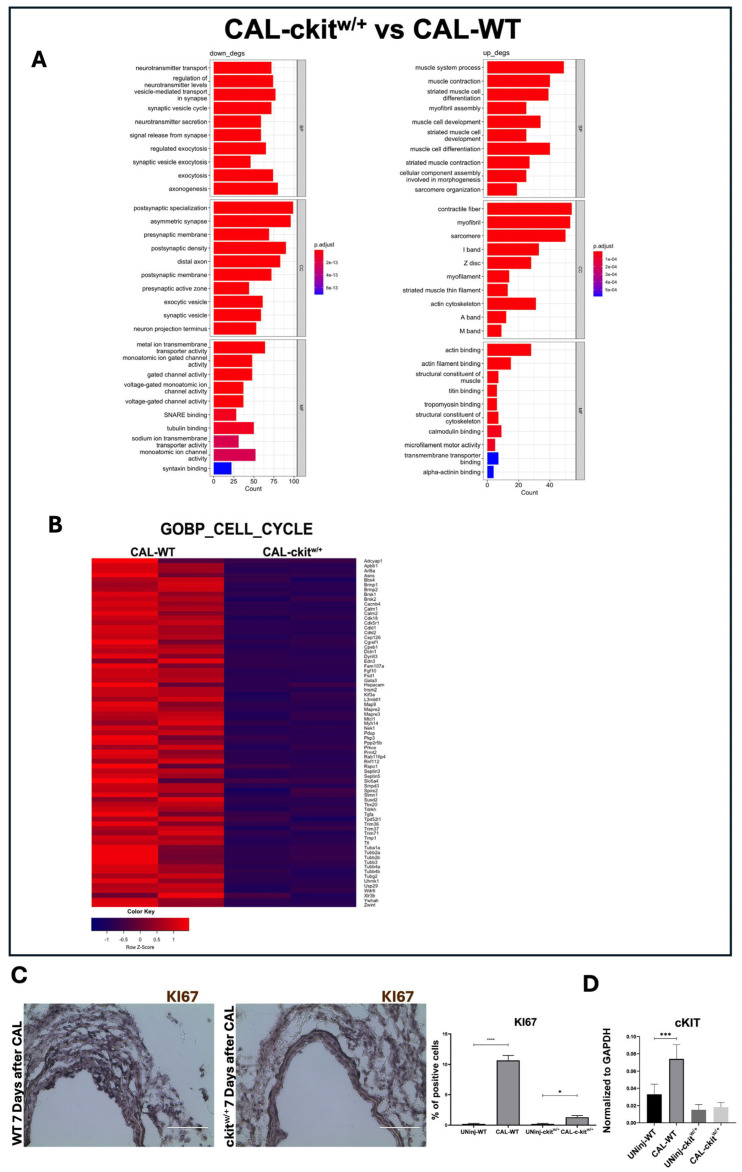
WT and c-kit^w/+^ mice display different global transcriptome profiles and gene-expression signatures in vivo after injury. (**A**) Gene ontology respectively showing the downregulated and upregulated pathways in the comparison in CAL-c-kit^w/+^ versus CAL-WT. (**B**) GSEA of G1 and G2 cell cycle-associated genes in CAL-c-kit^w/+^ versus CAL-WT. To perform RNA-Seq analysis, carotids were collected on Day-7: N = 6 WT and N = 6 c-kit^w/+^ carotids; contralateral carotids were used as controls; n = 3 carotids were pooled to create each of two biological replicates per group. (**C**) Representative light microscopy images and bar graph showing the percentage of Ki67-positive VSMC cells in carotid arteries from WT and c-kit^w/+^ mice 7 days after ligation compared with their respective controls. Scale bar = 100 μm (n = 3 technical replicates for each biological triplicate) **** *p* < 0.0001; * *p* = 0.0477. (**D**) Bar graph showing c-Kit expression at baseline and 28 days after ligation in UNinj-WT, CAL-WT, UNinj-c-kit^w/+^, and CAL-c-kit^w/+^ (n = 3 technical replicates for each biological triplicate). *** *p* = 0.0009. Data are the mean ± SD.

Direct comparison between CAL-c-kit^w/+^ and CAL-WT arterial samples revealed the significant downregulation of genes associated with muscle contraction, differentiation, and sarcomere organization in WT mice. In c-Kit haploinsufficient mice, the upregulated genes were linked to contractile fiber development and sarcomere structures such as the I band and Z disc, indicating a clear enhancement of molecular processes related to muscle contraction in CAL-c-kit^w/+^ compared with CAL-WT (Figure 7A). In contrast, CAL-WT arteries exhibited the upregulation of pathways involved in intracellular trafficking and metabolic exchange, reflecting active cell proliferation essential for the repair process (Figure 7A). Consistently, the GSEA of cell cycle-related genes demonstrated increased proliferative activity in CAL-WT compared with CAL-c-kit^w/+^ (Figure 7B).

Immunohistochemistry analysis further corroborated the RNA-Seq data. Indeed, at 7 days post CAL, injured carotid arteries from WT mice showed a significantly increased VSMC proliferation rate (ki67+VSMCs = 10 ± 2%) when compared with uninjured control WT arteries (ki67+VSMCs = 0.2 ± 0.1%) (Figure 7C). Also, in the CAL-c-kit^w/+^ mice, an increase in VSMC proliferation in carotid arteries 7 days post CAL was observed (ki67+VSMCs = 1.3 ± 0.3) compared with the uninjured control c-kit^w/+^ carotid arteries (ki67+VSMCs = 0.2 ± 0.1%) (Figure 7C). Nevertheless, VSMC proliferation in the CAL-c-kit^w/+^ carotid arteries was significantly lower than in the CAL-WT carotid arteries (Figure 7C). Importantly, in WT mice, c-Kit expression was found to be significantly upregulated in carotid sections after CAL compared with their uninjured counterparts (Figure 7D). This increase was instead poorly detectable in the c-kit^w/+^ samples (Figure 7D).

Overall, these results establish c-Kit as a key regulator of VSMC phenotypic switch upon vascular injury. Whereas WT-VSMCs downregulate contractile genes and adopt a proliferative program to mediate repair, c-Kit haploinsufficient SMCs remain locked in a contractile state and fail to proliferate, adopting a senescent phenotype. This identifies c-Kit as a pivotal determinant of vascular repair capacity.

### 3.5. c-Kit Haploinsufficient Mice Display a Reduced Neointima Formation In Vivo in a Mouse Model of Carotid Ligation

**Figure 8:**
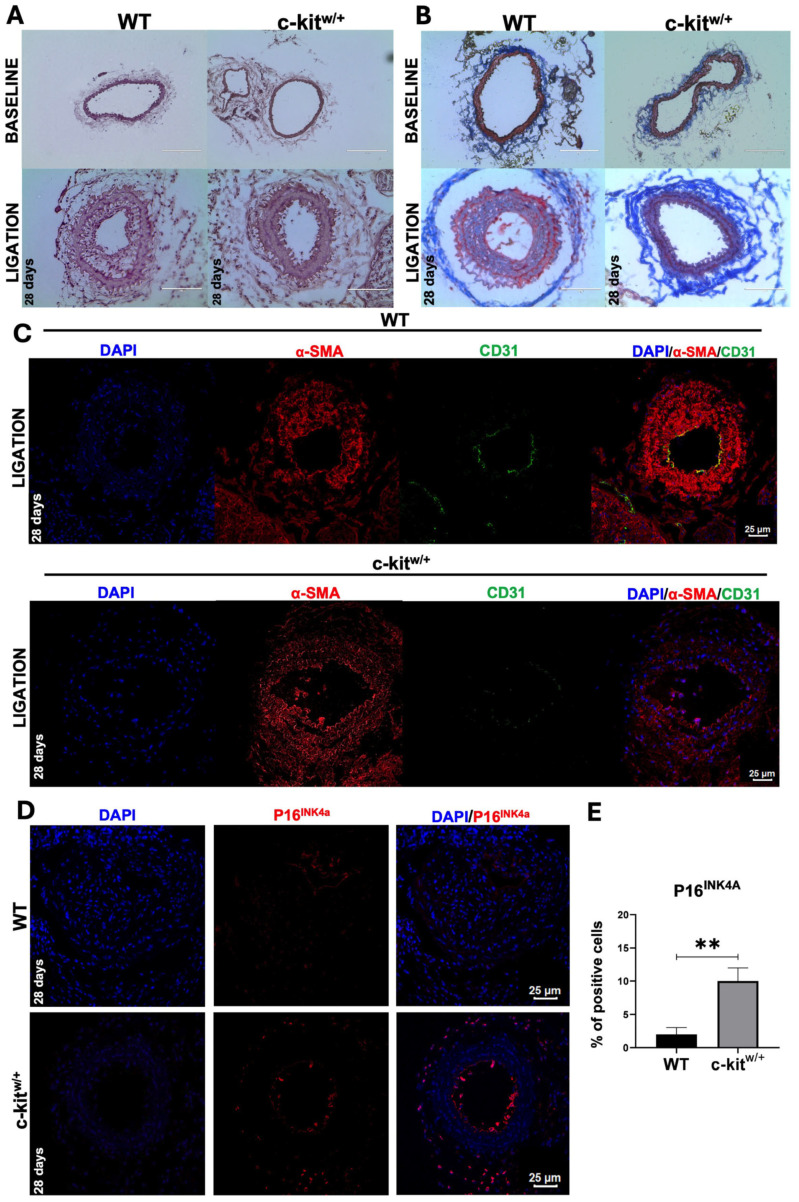
c-kit haploinsufficiency reduces SMC proliferation and increases cellular senescence after vascular damage in vivo. (**A**) Representative light microscopy images showing H&E (hematoxylin and eosin) on the carotid section at baseline and 28 days after CAL in WT and c-kit^w/+^ mice (representative of N = 3 mice per group). Scale bar = 200 μm (upper images) and 100 μm. (**B**) Representative Masson’s trichrome staining of carotid sections from WT mice compared with c-kit^w/+^ mice at the baseline and 28 days after CAL. Scale bar  = 200 μm (upper images) and 100 μm (representative of N = 3 mice per group). (**C**) Representative confocal images showing SMA and CD31 immunostaining on carotid sections from the WT and c-kit^w/+^ mice at the baseline and 28 days after CAL (α-SMA, red; CD31, green; nuclei, DAPI in blue). Scale bar = 25 μm (representative of N = 3 mice per group). (**D**,**E**) Representative confocal images and bar graph showing p16I^NK4a^-positive VSMCs in carotids from the WT and c-kit^w/+^ mice 28 days after ligation (p16^INK4a^, red; nuclei, DAPI in blue). Scale bar = 25 μm (N = 3 mice per group). ** *p* = 0.0034 vs. WT. Data are the mean ± SD.

A significant neointima formation was observed in WT mice 28 days after CAL (Figure 8A). In WT mice, neointima formation was accompanied by collagen deposition within the vascular lumen and the appearance of muscle fiber-like structures, two hallmark remodeling processes typically associated with vascular injury (Figure 8B). In contrast, the c-kit^w/+^ mice exhibited minimal neointima formation and collagen deposition (Figure 8A,B), consistent with our in vitro findings showing a reduced proliferative capacity of aortic c-kit^w/+^-SMCs (see Figure 1 and Figure 2).

Immunofluorescence staining on carotid sections 28 days after CAL showed that neointima was populated by α-SMA-positive SMCs in WT mice as well as in c-kit^w/+^ mice (Figure 8C). Remarkably, carotid sections obtained from WT mice at 28 days post CAL showed the presence of CD31-positive cells covering the neointimal lumen, a marker typically expressed by endothelial cells, pointing to the presence of endothelial-like structures and the progressive re-endothelialization of the neointima as part of the vascular remodeling process (Figure 8C). Contrariwise, CD31-positive cells were rarely found on the remodeled vessel lumen of injured c-kit^w/+^ mice carotid arteries (Figure 8C).

Finally, carotid artery sections collected 28 days after ligation revealed a significantly higher proportion of P16^INK4a^-positive VSMCs in c-kit^w/+^ mice compared with the WT controls (over 10% vs. <2%; Figure 8D,E). These findings were consistent with in vitro data from aortic SMCs isolated from both genotypes, supporting the hypothesis that a haploid level of c-Kit reduces the vascular SMC plasticity and proliferative capacity due to premature cellular senescence.

Overall, these findings show that c-Kit haploinsufficiency inhibits SMC proliferation and promotes a senescence-prone phenotype, thereby impairing vascular remodeling and reducing neointima formation—establishing c-Kit as a key driver of reparative SMC plasticity.

## 4. Discussion

This study identified c-Kit as a key regulator of reparative VSMC plasticity after vascular injury. By means of several complementary in vitro and in vivo approaches, we found that reduced c-Kit expression locks VSMCs in a hyper-contractile, senescence-prone state, blunts injury-induced cell-cycle activation, and limits neointima formation after arterial injury. Conversely, restoring c-Kit rescues both smooth-muscle differentiation and proliferative competence. These findings integrate and extend the prior literature on the role of c-Kit in vasculo-proliferative disorders.

In wild-type (WT) carotid arteries, injury suppressed contractile signatures and induced cell-cycle and trafficking programs, consistent with the canonical contractile-to-synthetic transition of VSMC phenotypic switch that enables arterial remodeling and repair [36]. c-Kit haploinsufficiency disrupted this transition at two levels: (i) primary c-kit^w/+^-VSMCs proliferated poorly and were refractory to platelet derived growth factor subunit B (PDGF-BB)-driven downregulation of the main SMC genes, and (ii) injured c-kit^w/+^ carotid arteries failed to coherently upregulate cell-cycle genes and instead acquired hyper-contractile gene expression. Together with the marked reduction in Ki67+ cells in vivo, these data strongly suggest that c-Kit operates upstream of the proliferative arm of the phenotypic switch. Prior reports have shown that c-Kit is activated after vascular injury and promotes VSMC migration/proliferation [18] and that c-Kit positive adventitial/perivascular cells contribute to neointima formation [37]. Our study adds mechanistic insights by linking c-Kit gene dosage to transcriptional control of the VSMC phenotypic switch and by demonstrating that inadequate c-Kit signaling preserves contractile identity at the expense of cell proliferation. The latter explains the robust reduction in neointima formation we observed at day 28 after carotid artery ligation (CAL).

A central finding of the present study is the tight association between reduced/low c-Kit and a senescent VSMC state. c-kit^w/+^-VSMCs showed increased Cdkn2a/Cdkn1a (p16^INK4a^/p21), elevated senescence-associated secretory phenotype (SASP) cytokines, and a progressive rise in p16^INK4a^-positive VSMCs with passaging; carotid arteries from c-kit^w/+^ mice contained more p16^INK4a^-positive cells both early and late after injury. Functionally, this c-Kit-deficit-dependent senescence program likely accounts for reduced BrdU incorporation in vitro and fewer Ki67+ cells and smaller neointima in vivo in c-Kit haploinsufficient mice. These data agree with work indicating that senescent VSMCs limit neointimal formation and restenosis by imposing a permanent cell-cycle arrest [37]. However, senescence is not uniformly beneficial. SASP factors can impair re-endothelialization, destabilize the extracellular matrix, and promote chronic inflammation [38]. Consistent with this, injured c-kit^w/+^ carotid arteries exhibited poor luminal CD31+ endothelial cell coverage at day 28, suggesting delayed re-endothelializations, a feature linked to thrombotic risk and plaque vulnerability. Thus, c-Kit insufficiency reduces neointima development through growth arrest but may create a pro-inflammatory, matrix-remodeling milieu that could compromise long-term arterial stability—a working hypothesis that fits with reports showing that senescent VSMCs and their SASP drive features of plaque progression and fragility in atherosclerosis [28,39,40]. Accordingly, the role of c-KIT in vascular remodeling seems context-dependent. In acute injury models, stem cell factor (SCF)/c-Kit signaling has been associated with VSMC proliferation and neointima growth [41]. In chronic atherogenesis, SMC-specific loss of c-Kit increased plaque burden, necrotic core size, and foam-cell–like features, whereas preserving c-Kit in SMCs limited disease progression [28]. Our data reconcile these observations by emphasizing dosage and timing: adequate c-Kit expression and signaling enable a controlled, transcriptionally coordinated VSMC switch that supports acute repair after injury and sustains VSMC proliferation and mitochondrial/metabolic competence while supporting endothelial regeneration. Inadequate c-Kit expression shifts the balance toward a senescence-dominant state—reducing neointima formation but fostering chronic inflammatory signaling and impaired endothelial repair that may accelerate atherosclerotic plaque progression.

Beyond effects on mature VSMCs, we show that c-Kit is required for adult progenitor commitment to the VSMC lineage. Wild-type cardiac stem/progenitor cells (CSCs) efficiently generated iVSMCs with the appropriate upregulation of smooth-muscle genes and a rise in c-Kit expression during commitment; c-kit^w/+^ CSCs rarely did so, and BAC-mediated normalization of c-Kit rescued both the differentiation and proliferative competence of iVSMCs in vitro. Although vascular adventitial/perivascular progenitors are the most proximate source of VSMCs after injury, CSCs provide a valid and informative model for several reasons. First, both CSCs and vascular progenitors have similar robust VSMC commitment and differentiation potential that engage shared signaling programs during VSMC specification [42,43]. Second, CSCs recapitulate key hallmarks of adult multipotent stem cell biology clonogenicity, self-renewal, and multi-differentiation potential while allowing genetic rescue at scale, which is technically more challenging in rare vascular progenitors. Third, our rescue experiment, feasible only in bona fide multipotent stem cells, shows c-Kit dosage as the causal variable, demonstrating that restoring c-Kit is sufficient to re-establish smooth-muscle differentiation.

We propose that c-Kit acts as a gatekeeper of reparative VSMC plasticity. With normal c-Kit expression, vascular injury triggers a coordinated transcriptional program: downregulation of contractile genes, induction of cell-cycle and trafficking pathways, metabolic reprogramming away from high mitochondrial dependence, temporary proliferation, matrix synthesis, and re-endothelialization support. When c-Kit is halved, VSMCs are blocked to a contractile/senescent state with robust SASP, insufficient proliferation, and reduced neointima, together with deficient endothelial regeneration. This model explains why c-Kit inhibition may reduce restenosis but risk maladaptive healing and why maintaining c-Kit in SMCs is protective in atherosclerosis.

## 5. Limitations

Our study had several limitations: the haploinsufficient model reduced c-Kit across all c-Kit expressing cell types (not SMC-specific); the CAL model lacked lipid-driven inflammation seen in atherosclerosis; and lineage tracing was not performed to identify and quantify progenitor contributions. Single-cell transcriptomics, SMC-restricted genetic perturbations in ApoE knock-out mice, and senescence-targeted interventions (senolytics) will be helpful to refine the c-Kit–senescence axis and its therapeutic window. Together, these data position c-Kit dosage as a gatekeeper of reparative VSMC plasticity—constraining senescence while enabling transient proliferation and progenitor-to-VSMC differentiation—to balance vascular repair against maladaptive remodeling.

## 6. Conclusions

In summary, this study identified c-Kit as a dosage-dependent regulator of vascular smooth muscle cell (VSMC) plasticity that orchestrates the balance between proliferation, differentiation, and senescence after vascular injury. By integrating transcriptional, cellular, and physiological data, we demonstrated that adequate c-Kit expression is essential for a coordinated contractile-to-synthetic transition, progenitor-to-VSMC differentiation, and effective arterial repair. These insights reconcile the prior, seemingly divergent literature across injury and atherosclerosis and suggest actionable strategies to fine-tune vascular repair while minimizing long-term vascular instability. We show that insufficient c-Kit signaling constrains reparative proliferation and promotes a senescence-prone, hyper-contractile phenotype. Conversely, restoring c-Kit expression reinstates both smooth-muscle differentiation and proliferative competence. These results underscore c-Kit’s role as a gatekeeper of reparative VSMC plasticity, integrating cell-cycle, metabolic, and transcriptional programs that enable adaptive vascular healing. From a translational standpoint, this work highlights a potential therapeutic window for “tuning” rather than permanently abolishing c-Kit signaling. Transient or locally targeted modulation of c-Kit activity may allow clinicians to balance restenosis prevention with the preservation of endothelial repair and plaque stability. Nonetheless, our study had several limitations. The mouse carotid ligation model reflects acute injury rather than chronic vascular disease; thus, future studies should test whether similar mechanisms operate in atherosclerosis or human vessels. Additionally, the downstream molecular mediators linking c-Kit to cell-cycle and SASP/senescence programs remain to be defined. Exploring these pathways could reveal new molecular targets to modulate VSMC plasticity and vascular aging.

In conclusion, we propose that c-Kit acts as a master regulator of reparative vascular remodeling, integrating injury signals to balance proliferation, senescence, and differentiation. These findings extend the current understanding of VSMC biology and open new avenues for the therapeutic modulation of vascular repair—emphasizing the importance of dosage and timing in maintaining arterial health.

## Data Availability

The original contributions presented in this study are included in the article/Supplementary Material. Further inquiries can be directed to the corresponding author.

## Supplementary Materials

The following supporting information can be downloaded at: https://www.mdpi.com/article/10.3390/cells14201641/s1, Supplementary Figure S1. (A) Principal component analysis (PCA) on the whole genome expression profile of carotids from uninjured and injured WT mice (UNinj-WT and CAL-WT, respectively) and uninjured/injured c-kit^w/+^ mice (UNinj-c-kit^w/+^ and CAL-c-kit^w/+^, respectively). (n = 3 carotids per group). (B) Heat map showing the expression of selected senescence markers in carotids from UNinj-WT vs. UNinj-c-kit^w/+^.
