# Supplementary material for: C-Kit Is Essential for Vascular Smooth Muscle Cell Phenotypic Switch In Vitro and In Vivo After Injury
Source: Cells. 2025 Oct 21;14(20):1641. doi: 10.3390/cells14201641 (PMC12564463; doi:10.3390/cells14201641)
Supplement: Supplementary file 1 [file cells-14-01641-s001.zip › cells-3927578-supplementary.pdf]

A

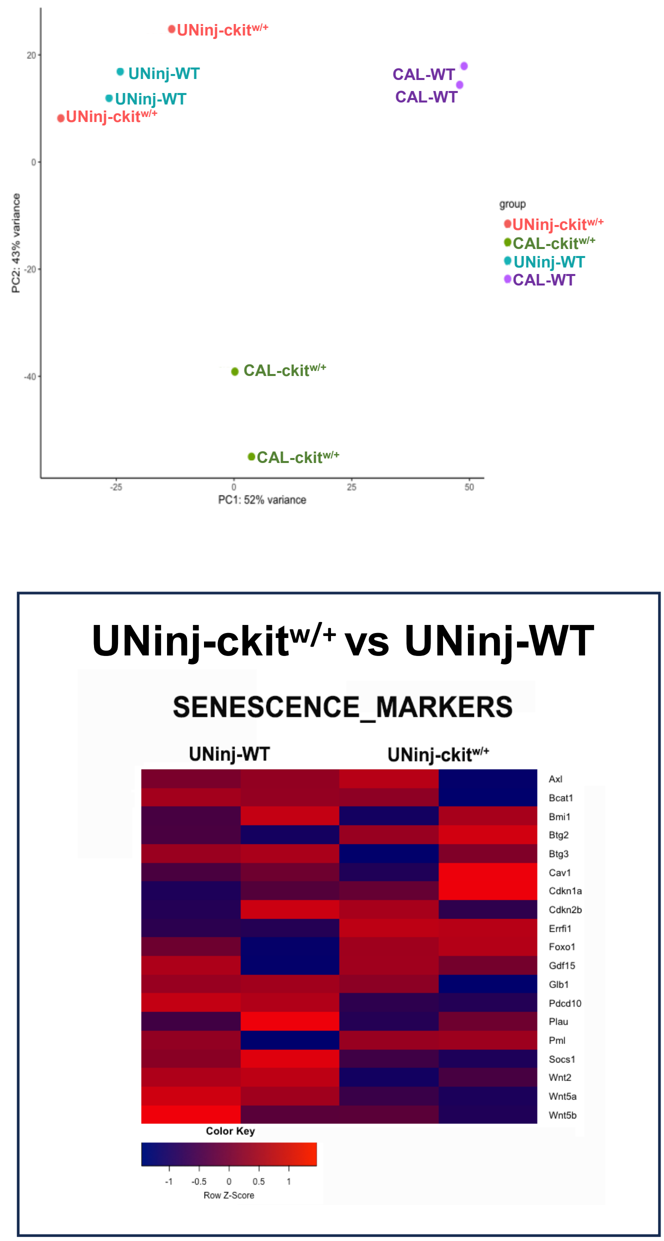

Supplementary Figure 1. (**A**) Principal component analysis (PCA) on the whole genome expression profile of carotids from uninjured and injured WT mice (UNinj-WT and CAL-WT, respectively) and uninjured/injured c-kit$^{w/+}$ mice (UNinj-c-kit$^{w/+}$ and CAL-c-kit$^{w/+}$, respectively). (n=3 carotids per group). (**B**) Heat map showing the expression of selected senescence markers in carotids from UNinj-WT vs UNinj-c-kit$^{w/+}$.
